# Lower limb conduit artery endothelial responses to acute upper limb exercise in spinal cord injured and able-bodied men

**DOI:** 10.14814/phy2.12367

**Published:** 2015-04-06

**Authors:** Julia O Totosy de Zepetnek, Jason S Au, David S Ditor, Maureen J MacDonald

**Affiliations:** 1 Department of Kinesiology, McMaster UniversityHamilton, Ontario, Canada; 2 Department of Kinesiology, Brock UniversitySt. Catharines, Ontario, Canada

**Keywords:** Endothelial function, exercise, flow-mediated dilation, shear rate, spinal cord injury

## Abstract

Vascular improvements in the nonactive regions during exercise are likely primarily mediated by increased shear rate (SR). Individuals with spinal cord injury (SCI) experience sublesional vascular deconditioning and could potentially benefit from upper body exercise-induced increases in lower body SR. The present study utilized a single bout of incremental arm-crank exercise to generate exercise-induced SR changes in the superficial femoral artery in an effort to evaluate the acute postexercise impact on superficial femoral artery endothelial function via flow-mediated dilation (FMD), and determine regulatory factors in the nonactive legs of individuals with and without SCI. Eight individuals with SCI and eight age, sex, and waist-circumference-matched able-bodied (AB) controls participated. Nine minutes of incremental arm-crank exercise increased superficial femoral artery anterograde SR (*P* = 0.02 and *P* < 0.01), retrograde SR (*P* < 0.01 and *P* < 0.01), and oscillatory shear index (OSI) (*P* < 0.001 and *P* < 0.001) in both SCI and AB, respectively. However, these SR alterations resulted in acute postexercise increases in FMD in the AB group only (SCI 6.0 ± 1.2% to 6.3 ± 2.7%, *P* = 0.74; AB 7.5 ± 1.4% to 11.2 ± 1.4%, *P* = 0.03). While arm exercise has many cardiovascular benefits and results in changes in SR patterns in the nonactive legs, these changes are not sufficient to induce acute changes in FMD among individuals with SCI, and therefore are less likely to stimulate exercise training-associated improvements in nonactive limb endothelial function. Understanding the role of SR patterns on FMD brings us closer to designing effective strategies to combat impaired vascular function in both healthy and clinical populations.

## Introduction

It is clear from in vitro*,* and more recently human studies, that alterations in both the magnitude and pattern of shear stress acting on the endothelial cell wall can have substantial influences on endothelial health. Elevations in anterograde shear rate (SR; a surrogate for shear stress in the absence of measures of blood viscosity) commonly observed with exercise are associated with increased cardiac output and muscle blood flow, and have been described as atheroprotective (Chiu et al. 2009; Chiu and Chien 2011). In contrast, it has been shown in vitro that low flow, oscillatory, or turbulent SR results in decreased endothelial cell function (Chiu et al. 2009; Chiu and Chien 2011).

Regular exercise has been shown to enhance endothelial function as evidenced by augmented flow-mediated dilation (FMD) of peripheral conduit arteries in humans (Clarkson et al. 1999). These enhancements may be partially mediated by the repeated increases in blood flow and SR that occur during exercise (Walther et al. 2008). Exercise not only leads to improvements in vascular structure and function in active regions (Tinken et al. 2010), but can also result in vascular adaptations in nonactive regions such as improved upper extremity FMD following leg cycling (Linke et al. 2001). Interestingly, recent work has reported larger acute releases in nitric oxide in response to oscillatory versus pure anterograde SR during exercise (Green et al. 2005). These findings support the concept that SR patterns may have important implications for endothelial health, and that acute stimulus response studies in this area may provide valuable information about regulation of endothelial health.

Several studies have investigated the effects of a single bout of lower body exercise on brachial artery FMD. Conflicting results from these studies include reports of acute increases (Harris et al. 2008; Zhu et al. 2010), decreases (Dawson et al. 2008; Johnson et al. 2012b) or no change (Rognmo et al. 2008; Jones et al. 2010) in brachial artery FMD following lower body aerobic or resistance exercise. Few of these studies have examined the components of the SR pattern (mean, anterograde, retrograde) during exercise and important information is likely missed when SR patterns induced by exercise are not considered or when only mean SR is reported.

Individuals with spinal cord injury (SCI) experience deconditioning of and augmented SR in paralyzed limb vasculature. Previous reports have shown reduced superficial femoral artery diameter, increased superficial femoral artery SR, but preserved or increased superficial femoral artery FMD in individuals with SCI compared to able-bodied controls (AB) (Schmidt-Trucksass et al. 2000; De Groot et al. 2003; de Groot et al. 2004; Thijssen et al. 2008). These unexpected paralyzed limb FMD responses after SCI could be attributed to chronic elevations in anterograde SR and subsequent upregulation of endothelial nitric oxide synthase, or perhaps explained by structural adaptations or neural factors (West et al. 2013). It is unknown whether upper extremity exercise, a common mode of exercise in this population, can induce acute alterations in SR in the nonactive lower extremities, and thereby lead to improvements in lower extremity FMD. Although anterograde SR is chronically elevated through sublesional vasculature, acute repeated increases in oscillatory SR from exercise (including both anterograde and retrograde) could create a stimulus for endothelial function adaptations. As such, individuals with SCI who lack lower limb function could benefit from these upper body exercise-induced increases in lower body SR and endothelial function improvements.

The objective of the present study was to investigate exercise-induced SR pattern changes in the superficial femoral artery during supine arm cranking in individuals with SCI and AB controls. Superficial femoral artery FMD was assessed before and after a single bout of arm-crank exercise. We hypothesized that in both groups arm-crank exercise would augment anterograde and retrograde SR in the superficial femoral artery and acutely improve superficial femoral artery FMD.

## Methods

### Participants

Eight individuals with SCI (level of injury T4–T11, AIS A-C, 11.9 ± 11.4 years postinjury) were recruited from Southern Ontario to participate. Eight AB persons matched for age, sex, height, body mass index, and waist circumference were recruited as controls (Table1). The study procedures were approved by the Hamilton Health Sciences Research Ethics committee in Hamilton, Ontario, Canada, adhered to the Declaration of Helsinki, and all of the participants gave previous written informed consent.

**Table 1:** Participant characteristics

Parameter	SCI	AB	*P*-value
Age, years	43 ± 7	43 ± 7	0.598
Height, m	1.8 ± 0.1	1.8 ± 0.1	0.540
Mass, kg	78.8 ± 18.2	86.2 ± 13.6	0.369
BMI, kg/m^2^	25.1 ± 4.2	25.8 ± 3.8	0.764
WC, cm	89.2 ± 14.5	91.9 ± 9.5	0.671
Body Fat, %	25.1 ± 6.4	20.7 ± 5.9	0.236
HR, bpm	66 ± 6	64 ± 14	0.674
Supine SBP, mmHg	127 ± 13	127 ± 13	0.962
Supine DBP, mmHg	73 ± 10	71 ± 8	0.672
Supine MAP, mmHg	91 ± 9	89 ± 9	0.809
VO_2_peak, mL/kg/min	22.3 ± 4.1	31.1 ± 5.5	0.009
PO_peak_, W	103.6 ± 17.8	129.4 ± 29.3	0.085

Values are mean ± standard deviation. SCI, spinal cord injury; AB, able-bodied; BMI, body mass index; WC, waist circumference; SPB, systolic blood pressure; DBP, diastolic blood pressure; MAP, mean arterial pressure; VO_2peak_, peak oxygen uptake; PO_peak_, peak power output. *P*-value refers to independent *t*-tests between groups (SCI vs. AB).

### Experimental design

Each participant came to the laboratory on two occasions for approximately one and a half hours. The first visit consisted of the peak aerobic capacity test (VO_2_peak) and body composition assessment. The second visit consisted of the vascular assessments and supine exercise. Participants abstained from caffeine and alcohol for ≥12 h and abstained from physical activity for a ≥24 h prior to both testing sessions. For the vascular assessments, participants arrived between 9–11 am in a fasted state (≥8 h), and assessments were conducted in a quiet, temperature-controlled room (22–24°C). Participants lay supine for 10–15 min prior to any data collection to ensure stability of resting measures. Heart rate was monitored continuously throughout all testing procedures using a single lead electrocardiograph (ECG, Model ML123, ADInstruments Inc., Colorado Springs, CO). Supine blood pressure was measured discretely in triplicate at baseline and immediately post exercise using an automated blood pressure device (Dinamap, GE Healthcare; Horten, Norway).

Superficial femoral artery FMD was assessed in the dominant leg (determined to be the same side as the dominant arm) before and after 9 min of continuous incremental supine arm-cranking exercise (increasing every 3 min with absolute intensities of 40, 50, 60 W for every participant).

### Visit 1 experimental procedures

#### Peak oxygen uptake

Peak oxygen uptake (VO_2_peak) was assessed via a symptom-limited graded arm-crank ergometer test (Lode Angio BV, Groningen, the Netherlands). Cardiac stability and heart rate were monitored throughout the test using a 1-lead ECG (PowerLab 15T, ADInstruments) and a Polar heart rate monitor (Polar T31, Polar Electro, Quebec, Canada). The test began with no resistance at a cadence of 60–80 rpm; after a 1-min warm-up the resistance increased every minute by 10 W. Participants continued arm cranking until volitional fatigue or if they were unable to maintain a cadence of 30 rpm. Expired gas and ventilatory parameters were acquired throughout the protocol. Blood pressure was assessed immediately following and throughout recovery to ensure that it returned to baseline values following the exercise test. VO_2_peak was determined to be the highest 30-sec average oxygen consumption.

#### Body composition

Dual energy x-ray absorptiometry (Hologic Inc., Waltham, MA) was utilized to assess for whole body fat (%). Dual energy x-ray absorptiometry is a “gold standard” measure of body composition and an effective method to characterize body composition in people with SCI (Jones et al. 1998).

### Visit 2 experimental procedures

#### Flow-mediated dilation and shear rate

To examine superficial femoral artery FMD, duplex ultrasound (Vivid Q, GE Medical Systems, Horten, Norway) was used to obtain a simultaneous brightness-mode image of the superficial femoral artery (13 MHz) and pulsed-wave blood velocity measurements (4 MHz). Superficial femoral artery images were acquired 3–5 cm distal to the common femoral bifurcation. Preocclusion data were collected for 30-sec followed by a 5-min period of ischemia via inflating a pneumatic cuff positioned on the distal thigh to an occlusion pressure of 200 mmHg (at least 50 mmHg above systolic blood pressure) using a rapid cuff inflator (E20 Rapid Cuff Inflator, AG 101 Cuff Inflator Air Source, Hokanson, WA). Upon cuff deflation, postocclusion data were collected continuously for 5-min.

Images were ECG-gated and obtained at a frame rate of 7.7 frames/s. Off-line analyses involved selecting end diastolic frames from preocclusion and postocclusion images and saving them to digital imaging and communications in medicine file format (Sante DICOM Editor, Version 3.1.20, Santesoft; Greece). End diastolic diameters were analyzed from the near wall to the far wall at the adventitia-media interface using custom-designed semiautomated edge-detection software (Artery Measurement System Image and Data Analysis, Tomas Gustavsson; Sweden). Relative FMD was then calculated as shown below:

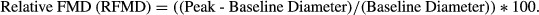


Test–retest reliability has been calculated previously for superficial femoral artery RFMD for both SCI and AB in our laboratory (unpublished data); the intraclass correlation coefficient and coefficient of variation was 0.90 and 9% for SCI superficial femoral artery RFMD and 0.95 and 3% for AB superficial femoral artery RFMD, respectively.

Sample volume (gate width) for the pulsed-wave velocity measures encompassed the entire superficial femoral artery lumen (from intima-to-intima) so that measurements of blood velocity represent a mean of the entire cross-sectional area of the superficial femoral artery. Raw blood velocity profiles were outsourced to a spectral analyzer (Neurovision 500M TCD, Multigon Instruments; Yonkers, NY). Intensity-weighted mean red blood cell velocity was fast Fourier transformed and acquired with an analog to digital data acquisition system for off-line beat-to-beat analyses (PowerLab 16/35 with LabChart 7 Pro, ADInstruments Inc.). SR was calculated (Parker et al. 2009) as shown below:

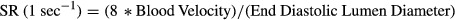


#### Exercise intervention

Prior to initiating exercise, baseline superficial femoral artery diameter and blood velocity data were collected. Mean SR, anterograde SR, retrograde SR, and oscillatory shear index (OSI) components were analyzed off-line using PowerLab. OSI represents a measure of the magnitude of shear oscillation or shear reversal; for purely oscillatory flow the OSI attains a maximum value of 0.5. Consistently high values of OSI have been associated with endothelial dysfunction (He and Ku 1996). OSI was calculated (Newcomer et al. 2008) as shown below:

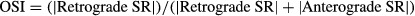


The vertical lines represent the absolute values of the SR components. Each participant then performed 9 min of continuous supine arm-crank exercise (3 × 3-min of increasing intensity: 40, 50, 60 W) using a Monark arm ergometer (Monark Rehab Trainer 881e, Monark Exercise AB, Varberg, Sweden). Participants were instructed to maintain a comfortable cadence of 60 rpm. During the last 30-sec of each exercise intensity, superficial femoral artery diameters and blood velocity measures (mean, anterograde, retrograde, OSI) were obtained. Exercise intensity and cardiovascular stability was maintained in these 30 sec of data collection, evidenced by a stable heart rate. Velcro straps were secured around the participant's hips and thighs to minimize lower body movement and to facilitate data collection, but were not tight enough to impact SR patterns. Prior to and following the 9-min exercise intervention, dominant leg RFMD (determined to be the same side as hand dominance) was assessed.

### Statistics

Statistical analyses were performed using SPSS 20.0 software (IBM Corporation, Armonk, NY). Baseline characteristics (demographics and vasculature) are presented as mean ± SD; independent *t*-tests were used to assess any differences between groups (SCI vs. AB). A factorial-repeated measures analysis of variance was used with the factor being group (SCI vs. AB) and the repeated measures being time (pre vs. postintervention) to determine the effects of arm exercise on superficial femoral artery RFMD. A post hoc *t*-test with Bonferroni correction was performed when a significant interaction effect was found. Paired *t*-tests were used to determine the differences in SR components (mean, anterograde, retrograde) at baseline at and end-intervention. Statistical significance was determined at *P* < 0.05.

If baseline diameter differences were found in response to the exercise intervention for RFMD assessments, covariate-adjusted means for diameter change using a linear mixed model taking into account repeated measures was used. Briefly, we logarithmically transformed pre- and peak-diameters and calculated the change in diameter on the logged scale. This value was entered as the dependent variable with log baseline as the covariate. Covariate-adjusted means for diameter change during the FMD assessments were obtained, back-transformed, and then converted to a ‘corrected’ adjusted percentage change by subtracting one from the back-transformed value and multiplying it by 100 (Atkinson et al. 2013). Data are presented as mean ± SD, with *P* < 0.05 considered statistically significant.

## Results

All participants completed the entire protocol. No differences were found in age, anthropometrics, % body fat, resting heart rate, or resting blood pressure between SCI and AB controls. Aerobic capacity (VO_2_peak) was lower in SCI (*P* < 0.01) (Table1). Heart rate and blood pressure at rest and in response to exercise are shown in Table2.

**Table 2:** Hemodynamic responses to exercise intervention. Heart rate and blood pressure of participants before and after exercise intervention. Data are for both persons with spinal cord injury and able-bodied controls

Parameter	Before	After	*P*-value
Spinal cord injury			
HR, bmp	62 ± 12	125 ± 17	<0.001
Supine SBP, mmHg	126 ± 10	133 ± 14	0.12
Supine DBP, mmHg	79 ± 7	69 ± 3	<0.01
Able-bodied			
HR, bmp	56 ± 11	113 ± 12	<0.001
Supine SBP, mmHg	124 ± 13	137 ± 16	<0.01
Supine DBP, mmHg	77 ± 13	70 ± 12	0.05

Values are mean ± standard deviation. HR, heart rate; SBP, systolic blood pressure; DBP, diastolic blood pressure pre- (before) and post- (after) exercise intervention. *P*-values refer to paired *t*-tests before and after exercise.

**Figure 1:**
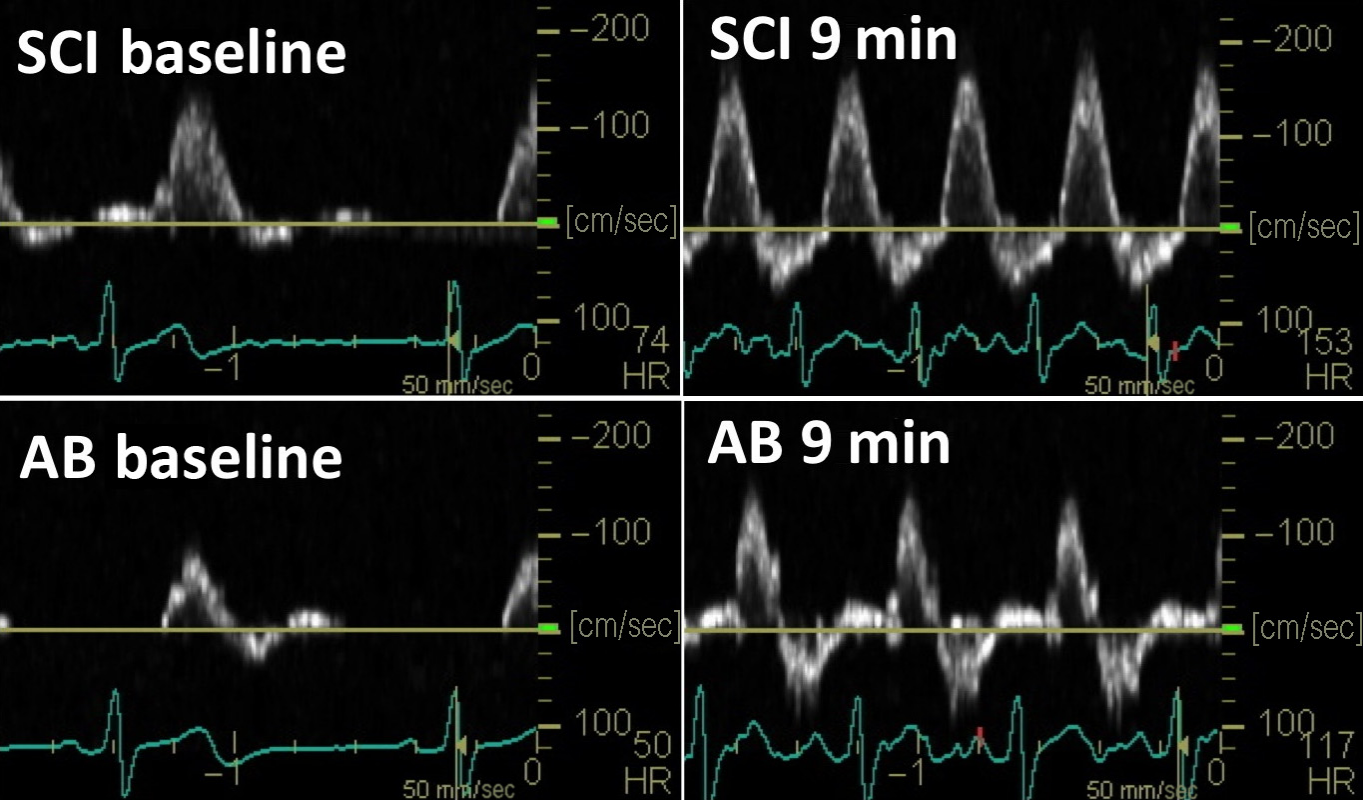
Arm-crank exercise intervention (Doppler Screen Capture). Superficial femoral artery blood velocity profiles at baseline and at 9-min of the arm-crank exercise intervention for an individual with spinal cord injury and an able-bodied control.

**Figure 2:**
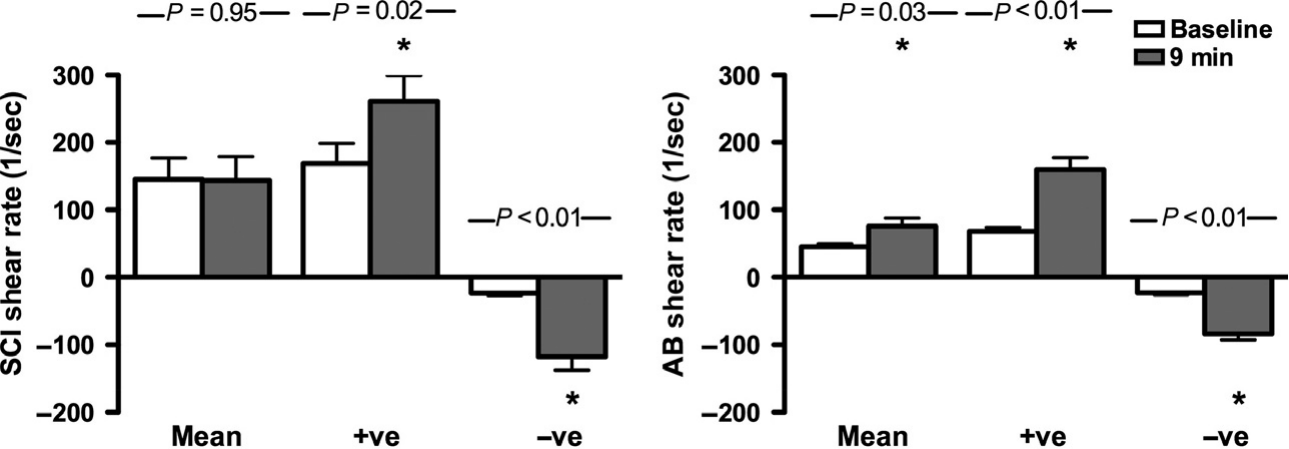
Shear rate responses to arm-crank exercise intervention. Mean, anterograde (+ve), and retrograde (−ve) shear rate patterns at baseline and at 9 min of arm-crank exercise for persons with spinal cord injury (SCI) and able-bodied controls (AB). Error bars represent standard deviation. *P-value < 0.05 baseline versus 9 min.

Doppler blood velocity profiles for superficial femoral artery at baseline and at 9 min of arm exercise in SCI and AB are shown in Figure1. Average heart rate was not different between groups at rest or at 9 min of exercise. These representative blood velocity profiles show enhanced anterograde SR in SCI with comparable retrograde SR between groups at both rest and at 9 min of exercise. Group averages for anterograde and retrograde SR at baseline and at 9 min of exercise are shown in Figure2.

**Figure 3:**
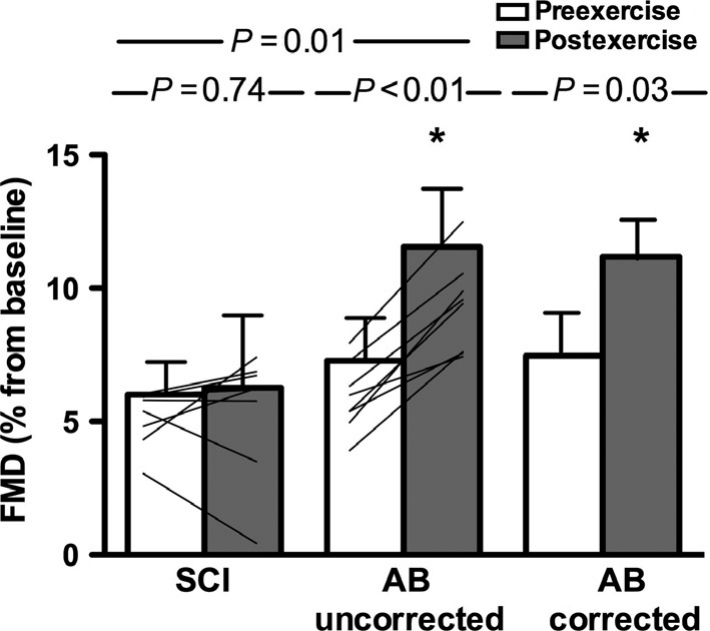
Relative flow-mediated dilation responses to arm-crank exercise intervention. Relative flow-mediated dilation (RFMD) before and after the arm-crank exercise intervention in SCI and AB; mean and individual data are presented. Corrected RFMD refers to covariate-adjustments for baseline diameter differences. Error bars represent standard deviation. *P-value < 0.05 preexercise versus postexercise.

Time between arm-crank exercise and post-RFMD assessment was 77 ± 19 and 75 ± 31 sec for SCI and AB, respectively (*P* = 0.84). During arm exercise, there were increases in the absolute magnitude of superficial femoral artery anterograde SR (*P* = 0.02), retrograde SR (*P* < 0.01), and OSI (*P* < 0.001) in the SCI group, but no change in RFMD post-exercise (*P* = 0.74). There were increases in the absolute magnitude of all superficial femoral artery SR components (mean SR *P* = 0.03, anterograde SR *P* < 0.01, retrograde SR *P* < 0.01, OSI *P* < 0.001) during arm exercise in the AB group, and an increase in RFMD post-exercise (*P* < 0.01) (Table3, Figs2 and 3). A difference in baseline diameter pre- to postexercise in the AB group was observed (Table3); after adjusting for baseline diameter the significant improvement in RFMD for the AB group remained (*P* = 0.03) (Fig.3). When looking at SCI versus AB, comparable changes in SR parameters were observed with exercise, but smaller FMD changes were observed in SCI versus AB.

**Table 3:** Vascular responses to exercise intervention. Superficial femoral artery flow-mediated dilation and shear rate characteristics of participants before and after exercise intervention. Data are for both persons with spinal cord injury and able-bodied controls

Parameter	Before	After	*P*-value
Spinal Cord Injury			
EDLD, mm	5.62 ± 0.58	5.51 ± 0.54	0.693
Peak SR, s^−1^	705 ± 325	681 ± 401	0.718
AUC_SR_, 10^3^	23.4 ± 15.7	23.5 ± 21.7	0.988
OSI	0.15 ± 0.08	0.31 ± 0.10	<0.001
Able-Bodied			
EDLD, mm	7.45 ± 0.90	7.12 ± 0.93	<0.001
Peak SR, s^−1^	633 ± 123	636 ± 143	0.943
AUC_SR_, 10^3^	22.8 ± 15.8	29.4 ± 16.5	0.213
OSI	0.25 ± 0.05	0.35 ± 0.04	<0.001

Values are mean ± standard deviation. EDLD, baseline end diastolic lumen diameter; SR, shear rate; AUC_SR_, shear rate area under curve up to maximum artery dilation during pre- (before) and post- (after) intervention flow-mediated dilation; OSI, oscillatory shear index. *P*-values refer to paired *t*-tests before and after exercise intervention.

## Discussion

While 9 min of arm-cranking exercise resulted in increases in both anterograde and retrograde SR through the nonworking limb (superficial femoral artery) in both SCI and AB, superficial femoral artery RFMD was only augmented in the AB group. These group differences in nonactive limb FMD responses to acute exercise indicate that arm exercise training is not likely to improve superficial femoral artery function in individuals with SCI. These findings highlight the multilayered regulation of endothelial function and the requirement to consider many factors including SR magnitude and pattern as well as metabolic and neural influences when designing interventions to impact endothelial function.

When assessing individual RFMD responses it appears that some participants with SCI were ‘responders’ while others were ‘non-responders’ to the exercise intervention (i.e., exercise caused an improvement in nonactive limb RFMD) (Fig.3). A closer look at participant characteristics of ‘responders’ versus ‘non-responders’ revealed no particular pattern for age, level or severity of injury, years postinjury, blood pressure, smoking, anthropometrics, or baseline RFMD. The unchanged RFMD in the SCI group after arm-crank exercise may be associated with: (1) lack of exercise-induced increases in systolic blood pressure; (2) existence of a critical threshold of SR change or ratio of anterograde to retrograde SR change required to induce changes in RFMD; (3) elevated systemic oxidative stress; and/or (4) lack of sympathetic nervous system activity.

Previous work in humans has suggested acute endothelial changes may require a critical threshold of exercise intensity, likely related to increases in blood pressure and SR (Hallmark et al. 2014). Although the specific exercise intensity threshold required for RFMD changes is unknown, it has been proposed that RFMD increases following moderate intensity exercise (Johnson et al. 2012a), and decreases following high intensity exercise (Rognmo et al. 2008; Birk et al. 2013). In our study, both the SCI and AB groups exercised at the same power output resulting in the groups exercising at 60 and 46% of their peak power output, respectively. These intensity levels are both considered moderate, so the inconsistent RFMD response is more likely to be attributed to group differences in blood pressure and SR with exercise. The AB group experienced exercise-associated increases in systolic blood pressure and heart rate while the SCI group experienced increases in heart rate only. It has been demonstrated that exercise-induced shear stress-mediated nitric oxide release depends on increases in both blood pressure and heart rate, and cannot be achieved with increases in heart rate alone (Green et al. 2002).

With regards to SR, it has been shown that elevating the brachial artery retrograde SR component during lower body exercise above normal physiological levels attenuated brachial artery RFMD (Johnson et al. 2012a). In the present study the SCI group experienced relatively greater magnitude increases in the retrograde component of SR (400 vs. 265% increase), less anterograde SR (55 vs. 135% increase), and more OSI (203 vs. 43%) compared to AB. Perhaps the lower relative anterograde increases and higher OSI change with exercise in the SCI group resulted in less atheroprotective SR alteration on the endothelial cells. An alternative explanation may be linked to the higher baseline anterograde SR observed in the SCI group (*P* < 0.01); it is possible that when exercising at a moderate intensity the SCI group achieved an absolute anterograde SR level close to what is experienced during high intensity exercise in AB. The consequence of these different SR pattern responses between groups could have contributed to the differential FMD changes observed between groups.

The pathophysiology of SCI is characterized by increased oxidative stress secondary to the primary injury (Jia et al. 2012). In addition, reactive oxygen species production is greater in obese compared to lean adults (Vincent et al. 2004), and in the present study the SCI cohort was obese (body fat >25%) while the AB cohort was not (body fat 21%) (Table1). Reactive oxygen species production can scavenge nitric oxide and thereby impair nitric oxide-dependent vasodilation, and in turn, RFMD (Jessup 1996). Exercise is also known to increase reactive oxygen species (Gomes et al. 2012) and can reduce nitric oxide bioavailability (Bergholm et al. 1999); elevations in reactive oxygen species have been suggested as a potential mechanism for the observed reduction in RFMD immediately following high intensity exercise (Birk et al. 2013). It is probable that individuals with SCI have higher levels of basal oxidative stress, and may respond to exercise by producing a greater quantity of reactive oxygen species when compared to AB controls. This increased oxidative stress with exercise may have contributed to the unchanged superficial femoral artery RFMD in the SCI group in the present study.

The final factor to take into consideration when examining the lack of RFMD response to exercise in the SCI group is sympathetic activity. Since sympathetic nerve activity decreases for several hours following an exercise bout (Floras et al. 1989; Halliwill et al. 2013), it is possible that improvements in RFMD following a single bout of moderate intensity exercise can be partially explained by attenuation of sympathetic flow to conduit artery smooth muscle, in addition to increased nitric oxide bioavailability in AB persons. It is likely that sympathetic innervation to the peripheral vasculature was altered in all participants with SCI in the present study; evidence of altered sympathetic activity in the SCI group is seen when examining superficial femoral artery diameter responses to the exercise intervention. In the AB group, superficial femoral artery diameter decreased during the exercise intervention (Table3) providing an indication of supra-spinal sympathetic regulation. No changes in superficial femoral artery diameter were seen in the SCI group. In a state of altered sympathetic modulation such as SCI, it is conceivable that the lack of change in sympathetic tone postexercise could contribute to the absence of RFMD change.

### Limitations

Several limitations need to be addressed. We did not exclude participants with SCI with previous cardiovascular disease or current cardiovascular risk factors; five individuals with SCI were previous or current smokers, and one had sustained a previous stroke. None of the AB participants were smokers or had previous or current cardiovascular disease. However, previous work in our laboratory observed acute improvements in brachial FMD in individuals with coronary artery disease following exercise (Currie et al. 2012). Another limitation is that all participants in both the SCI and AB groups exercised at the same absolute intensity resulting in slightly different relative intensities; however, the intensity levels were both considered moderate. The AB group was working at a lower relative intensity yet achieved a greater relative anterograde SR response and improved RFMD; therefore the absence of response in the SCI group could not be due to the different relative intensities. Further, increased muscular stabilization of the legs and torso (seen in the AB group) has been shown to increase oxygen uptake during arm-crank exercise (Sawka 1986); therefore the relative oxygen consumption of the muscles responsible for arm cranking between groups was likely more similar. A final limitation in the present study was the lack of any direct examination of sympathetic nervous system activity, such as the sympathetic skin response test demonstrated to be an appropriate estimate of autonomic function in SCI (Berger et al. 2014). Knowledge of sympathetic innervation at rest and during the exercise intervention could provide insight on the distinct responses between SCI and AB.

### Summary and future directions

The SCI and AB cohorts responded differently to arm exercise-induced superficial femoral artery SR alterations: the SCI group experienced no change in superficial femoral artery RFMD while the AB group had a significant improvement in superficial femoral artery RFMD. Upon closer inspection of SR patterns between groups, we found that the SCI group had significantly higher resting anterograde SR (*P* < 0.01), and in response to exercise the SCI group experienced smaller relative anterograde (atheroprotective) and a greater relative retrograde (atherogenic) SR increases when compared to AB. Perhaps baseline and exercise SR patterns influence endothelial cell responses. It is likely that altered metabolic (elevated reactive oxygen species) and neural (disrupted sympathetic innervation) factors contribute to the way in which endothelial cells respond to altered SR environments.

It is possible that in SCI, arm-only exercise does not provide a sufficient stimulus to influence peripheral vessels that lack sympathetic innervation. In contrast, perhaps lower extremity exercise (i.e., functional electrical stimulation, body weight supported treadmill training, combined arm-leg exercise) could evoke upper extremity vascular function improvements. Future studies should explore the short and long-term effects of different modes, intensities, and durations of exercise on endothelial health in both healthy and clinical populations, as well as resolve the influence of metabolic and neural factors on endothelial function responses.
